# Phyloproteomic study by MALDI-TOF MS in view of intraspecies variation in a significant homogenous phytopathogen *Dickeya solani*

**DOI:** 10.1038/s41598-023-46012-3

**Published:** 2023-11-01

**Authors:** Agata Motyka-Pomagruk, Weronika Babinska-Wensierska, Wojciech Sledz, Anna-Karina Kaczorowska, Ewa Lojkowska

**Affiliations:** 1 Laboratory of Plant Protection and Biotechnology, Intercollegiate Faculty of Biotechnology University of Gdansk and Medical University of Gdansk, University of Gdansk, 58 Abrahama, 80-307 Gdańsk, Poland; 2 Research & Development Laboratory, Intercollegiate Faculty of Biotechnology University of Gdansk and Medical University of Gdansk, University of Gdansk, 20 Podwale Przedmiejskie, 80-824 Gdańsk, Poland; 3 Collection of Plasmids and Microorganisms (KPD), Faculty of Biology, University of Gdansk, 59 Wita Stwosza Street, 80-308 Gdańsk, Poland

**Keywords:** Pathogens, Applied microbiology, Food microbiology, Infectious-disease diagnostics, Mass spectrometry

## Abstract

*Dickeya solani* is an economically significant pectinolytic phytopathogen belonging to the *Pectobacteriaceae* family, which causes soft rot and blackleg diseases. Despite its notable impact on global potato production, there are no effective methods to control this pest. Here, we undertook a phyloproteomic study on 20 *D. solani* strains, of various origin and year of isolation, with matrix-assisted laser desorption/ionization time-of-flight mass spectrometry (MALDI-TOF MS) supported by an in-depth characterization of the strains in terms of the virulence-associated phenotype. In spite of high homogeneity in this species, we herein revealed for the first time intraspecies variation in the MALDI-TOF MS protein profiles among the studied *D. solani* isolates. Finally, representative mass spectra for the four delineated clades are presented. A majority of the analysed *D. solani* strains showed high virulence potential, while two strains stood out in their growth dynamics, virulence factors production and ability to macerate plant tissue. Nonetheless, the metabolic profiles of *D. solani* strains turned out to be uniform, except for gelatinase activity. Given that all *D. solani* isolates distinctly grouped from the other *Dickeya* species in the MALDI-TOF MS analysis, there is strong evidence supporting the potential routine use of this method for fast and reliable to-species identification of *D. solani* isolates of environmental origin.

## Introduction

*D. solani* are Gram-negative, pectinolytic, facultatively anaerobic rods belonging to the *Pectobacteriaceae* family (former *Erwinia* genus), which currently encloses 13 *Dickeya* spp. and 21 *Pectobacterium* spp.^1^. Soft rot *Pectobacteriaceae* (SRP) are the causative agents of soft rot on many economically important plants in addition to blackleg restricted to potato (*Solanum tuberosum* L.). These phytopathogens differ in their host specificity as several species, like *Pectobacterium carotovorum*, *Pectobacterium odoriferum*, *Pectobacterium versatile* or *D. dadantii* exhibit a broad host range involving multiple crops, vegetables and ornamental plants. However, *D. solani* and *P. atrosepticum* strains have been isolated predominantly from *S. tuberosum*^2^.

In general SRP infiltrate into the progeny tubers from the infected mother tuber^3^. The contaminated soil, water, alternative hosts or agricultural machinery may also contribute to SRP dispersal^4^. Once SRP reach the susceptible host, bacterial cells colonize the plant parenchyma and xylem vessels to set up a systemic infection. Aggregation of the pathogenic cells in the form of biofilm or emboli within the xylem results in clotting of the vessels, which leads to wilting and rotting of the whole plant^3^. In the case of soft rot on potato tubers, the parenchymatous tissue gets water-soaked and macerated, blackens upon contact with air and develops an unpleasant odour due to secondary infections caused by anaerobic bacteria and fungi^5^. The soft rot disease symptoms are present in the farmlands, during storage, transportation or marketing of the crop, while blackleg is recognized solely in the fields.

The soft rot and blackleg diseases have a significant impact on the potato production sector. For instance in the Netherlands, the downgraded or rejected seed potato tubers accounted for € 12 M losses each year from 2003 to 2007^6^. The total damage to the European potato sector was estimated to reach € 46 M each year; however, high differences between the years were noticed^1^. In spite of the high economic significance of SRP, there is a lack of available control methods^7^, which underlines a substantial need for reliable diagnostic and to-species identification techniques. At present, these phytopathogens are classified by cultural, serological, fatty acid methyl ester (FAME) in addition to PCR-based methods^4^. As matrix-assisted laser desorption/ionization time-of-flight mass spectrometry (MALDI-TOF MS) was proven successful in fast and accurate species identification in terms of human pathogens isolated from the clinical settings^8^, we anticipate high potential and applicability of this technique for the proper classification and typing of bacterial phytopathogens. Furthermore, the data gathered by Caputo et al.^9^ in their study on *S. epidermidis* strains emphasize the capability of MALDI-TOF MS to distinguish between strains exhibiting specific phenotypic features associated with the strain pathogenicity, such as biofilm formation. This discrimination was achieved through the use of an in-house reference library containing main spectra profiles of strains that are definitively known to possess or lack a particular feature. In the case of bacterial plant pathogens, it is pivotal to accurately diagnose *D. solani*-caused infections as this pathogen has efficiently spread since its first identification in 2004–2005 to diverse climates and even distant geographical locations^10–12^. Notably at higher temperatures, *D. solani* tends to be more virulent on potato than the other *Dickeya* spp.^10,13^. Due to the aggressiveness and invasiveness of this pest, Scotland has implemented a ‘zero tolerance’ approach to the Scottish Seed Potato Classification Scheme^10^, while Israel, Jamaica and Northern African countries considered all *Dickeya* spp. as quarantine microorganisms^1^.

The destructive action of SRP is associated with the effective production of virulence factors directly at the infection site. Once high local bacterial cell density is reached, the *quorum sensing*-dependent expression of virulence-associated determinants is turned on^14^. As the invading pathogen needs to tackle the plant cell wall on its way to assimilable nutrients, crucial importance in the pathogenesis of SRP has been attributed to the production and secretion of plant cell wall degrading enzymes (PCWDEs) *i.e.* pectinases, cellulases and proteases^15^.

Indeed, pectinases play a significant role in development of characteristic maceration symptoms by SRP. Pectate lyases (Pels), cutting the α-1,4 glycosidic bonds, are considered the major pectin-degrading enzymes^16^. Regarding *D. solani*, this bacterium produces 9 endo-pectate lyases, *i.e.* PelA-E, Pel I, PelL, PelN and PelZ, 1 rhamnogalacturonate lyase RhiE, 2–3 exo-polygalactronases PehVWX, an endo-polygalacturonase PehN, a predicted pectin lyase PnlG in addition to one pectin acetyl esterase PaeY and two pectin methyl esterases *i.e.* PemA and PemB^17^.

Furthermore, *Dickeya* spp. secrete one cellulase CelZ via the Type II Secretion System^17^. Strong evidence for the involvement of cellulases in the pathogenicity of SRP was given by Walker et al.^17,18^. *D. solani* displays also proteolytic activity associated with the production of four metalloproteases (PrtG, PrtA, PrtB, PrtC)^17^. Interestingly, the contribution of proteases to the overall pathogenicity of SRP is a subject of an ongoing debate^19–21^. For instance, *Dickeya* spp. EC16 mutants, lacking proteolytic activity, triggered disease symptoms as efficiently as the wild-type strain, not only on potato tissue, but also on chrysanthemum^19^. On the other hand, Potrykus et al.^20^ identified a single *D. solani* strain incapable of proteases production that showed also impaired ability to macerate potato tissue.

In terms of the other pathogenicity-related features of SRP, the growth phase and the final population density were linked before with the virulence of SRP^14^. Also, providing an insight into the metabolic profile of the pathogen proved useful in relation to studying its virulence potential^14^. Concerning crucial metabolic requirements of SRP, iron is an essential cofactor, not only for ATP synthesis, but also for the establishment of bacterial virulence^22^. To sequester this element, *Dickeya* spp. produce and secrete siderophores, chrysobactin and achromobactin^23^. Also, swimming motility was associated with bacterial virulence^24,25^, in particular at an early stage of infection. Due to the production of extracellular polysaccharides (EPS) and *e.g.* adhesins, bacterial cells attach and aggregate within the 3D biofilm structures. Such a colonization strategy allows for efficient invasion of the pathogen within the plant tissue in addition to facilitating bacterial survival under an unfavourable environment^26,27^.

Previous works reported high genomic homogeneity among *D. solani* strains by applying average nucleotide identity (ANI), DNA–DNA hybridization (DDH), multilocus sequence analysis (MLSA), pulsed field gel electrophoresis (PFGE)^11^, restriction fragments length polymorphism-PFGE (RFLP-PFGE), repetitive PCR (rep-PCR) with ERIC, BOX or REP primers^28^, BRIG-^29^ or MAUVE-based^30^ whole genome comparisons in addition to core-genome phylogeny^29^. Contrarily, Potrykus et al.^20^ and Golanowska et al*.*^28^ observed differences between *D. solani* isolates in the virulence-associated phenotypes in addition to the ability to macerate plant tissue. In this view, we undertook a first large-scale phyloproteomic study on 20 *D. solani* strains with MALDI-TOF MS. We aimed not only at investigating the variability in the whole proteome among a vast pool of *D. solani* strains of diverse origin and year of isolation, but also at the assessment of the applicability of MALDI-TOF MS for routine identification of *D. solani* isolates. In addition, biodiversity among the tested *D. solani* strains in terms of the most important virulence-associated features, *i.e.* plant tissue macerating potential, growth rate, PCWDEs activity, motility, biofilm formation, siderophores production and metabolic profiles is shown. The herein presented data provide support for the applicability of MALDI-TOF MS for accurate, reliable and fast identification of *D. solani*, especially taking into consideration the lack of control methods and the dominating reliance on preventive measures.

## Methods

### Bacterial strains

The included *D. solani* strains (Table 1), as well as, the reference strains, *i.e. D. dadantii* 3937 in addition to potent biofilm producers *Pseudomonas aeruginosa* PAO1 and *Pseudomonas fluorescens* CCM2115, belong to the collection of phytopathogenic bacteria of the Intercollegiate Faculty of Biotechnology University of Gdansk and Medical University of Gdansk (IFB UG & MUG), Poland.

**Table 1:** *D. solani* strains analysed in this study.

Ordinal number	IFB collection number	Nos. from other collections	Country and year of isolation	Plant host	Literature reference
1	IFB0099^b^	IPO2276, LMG28824, 101A9/2005	Poland, 2005	Potato, cv. Santa	^44^
2	IFB0102	101A10/2005	Poland, 2005	Potato, cv. Santa	^44^
3	IFB0123	IPO2222^TS^	The Netherlands, 2007	Potato, cv. Melody	^11,44^
4	IFB0130	08.23.3.1A, IPO3337	France, 2008	Potato	^48^
5	IFB0167^b^	94A/10/2009	Poland, 2009	Potato, cv. Fresco	^12^
6	IFB0212^b^	1A/1/2010	Poland, 2010	Potato	^28^
7	IFB0223^b^	457, A5350	Germany, 2005	Rhizosphere of potato	^20^
8	IFB0231^b^	VIC-BL-25	Finland, 2008	Potato, cv. Victoria	^49^
9	IFB0240	SRG2-22	Finland, 2008	Potato, cv. Challenger	^49^
10	IFB0311^b^	132A/3/37/2011	Poland, 2011	Potato, cv. Innovator	^12^
11	IFB0417^b,c^	2.5	Portugal, 2012	Potato	^29^
12	IFB0421^b,c^	3.4	Portugal, 2012	Potato	^29^
13	IFB0455^a^	IPO3204	Israel, 2007	Potato, cv. Voyager	^50^
14	IFB0458	152/2013, 158A/1/2013	Poland, 2013	Potato, cv. Bellarosa	^51^
15	IFB0484	LMG25865, GBBC 2040, GBBC1512	Belgium, 2007	Potato, cv. Première	^52^
16	IFB0487^b^	151/2013, 148A/2/28/2013	Poland, 2013	Potato, cv. Vineta	^51^
17	IFB0695^b^	2/2014, 14A/2014	Poland, 2014	Potato, cv. Arielle	^29,51^
18	IFB0697	37/2014, 15A/(1)/2014	Poland, 2014	Potato, cv. Zuzanna	^51^
19	IFB0698	5/2014, 45A/(2)/2014	Poland, 2014	Potato, cv. Bellarosa	^51^
20	IFB0699	19/2014, 43A/(2)/2014	Poland, 2014	Potato, cv. Bellarosa	^51^

^a^Isolated from seed tubers exported from the Netherlands to Israel.

^b^*D. solani* strains subjected to whole genome sequencing and pangenome analysis in our former studies^28,29^. The corresponding full genomic sequences are publicly available in the GenBank database under the following accession numbers: CP024711, CP051457, JABAON000000000, CP024710, CP051458, JABAOO000000000, CP051459, CP051460, JABAOP000000000, JABAOQ000000000, respectively.

^c^These *D. solani* strains were isolated and identified at IFB UG & MUG from potatoes exported from Poland and grown in Portugal.

The bacterial strains (Table 1) were isolated at IFB UG & MUG from samples of potato plants collected from seed potato plantations either by the Polish Plant Health and Seed Inspection Service or employees of IFB UG & MUG. All these institutions possess permissions for the collection of cultivated potato crops, and the conducted experimental procedures complied with Polish and international guidelines and legislation. The resultant bacterial isolates were identified as *D. solani* at IFB UG & MUG using the methods described previously^31^. The *D. solani* strains isolated in other countries (Table 1) were provided by our collaborators, *i.e.* Dr. Jan van der Wolf from Wageningen Plant Research (The Netherlands), Dr. Yeshitila Degefu from Natural Resources Institute Finland (Finland), Dr. Johan Van Vaerenbergh from Flanders Research Institute for Agriculture, Fisheries and Food (Belgium) and Dr. Leah Tsror from Agricultural Research Organization—The Volcani Institute (Israel), or acquired from The International Center for Microbial Resources (France) or Belgian Coordinated Collections of Microorganisms (Belgium).

The full genomic sequences of IFB0099, IFB0167, IFB0212, IFB0223, IFB0231, IFB0311, IFB0417, IFB0421, IFB0487 and IFB0695 *D. solani* strains, have been deposited in the GenBank database^29,30^ and are publicly available under the following accession numbers CP024711, CP051457, JABAON000000000, CP024710, CP051458, JABAOO000000000, CP051459, CP051460, JABAOP000000000, JABAOQ000000000, respectively.

### MALDI-TOF MS analysis

#### Sample preparation and acquisition of MALDI-TOF MS spectra

All the *Dickeya* spp. strains were grown on TSA plates (GRASO Biotech, Poland) at 28 °C for 48 h. To ensure high quality of the MALDI-TOF MS spectra, the ethanol/formic acid (EtOH/FA) extraction procedure was performed during sample preparation according to the guidelines of the MALDI Biotyper system manufacturer (Bruker Daltonics GmbH, Bremen, Germany). Fresh bacterial colonies were picked with an inoculation loop and suspended in 300 μl of HPLC-grade water using a vortex. Subsequently, 900 μl of absolute EtOH were added, the samples were mixed thoroughly and centrifuged at 13,000 rpm for 3 min. The supernatant was discarded, and the remaining cell pellet was centrifuged for a second time to remove ethanol residues and then air-dried at room temperature for 5–10 min. To each sample, 25 μl of 70% [v/v] formic acid was added to dissolve the dried cell pellet prior to mixing by pipetting. Next, an equal volume of acetonitrile was added, mixed carefully, and centrifuged for 3 min. One μl of each supernatant was transferred onto an MSP 96 reusable polished stainless steel plate with 96 positions (spots) (Bruker Daltonics GmbH, Bremen, Germany) and air-dried at room temperature. Finally, the spots were overlaid with 1 μl of the freshly prepared HCCA (α-Cyano-4-hydroxycinnamic acid; Bruker Daltonics GmbH, Bremen, Germany) matrix solution (HCCA dissolved in 50% [v/v] acetonitrile, 47.5% water and 2.5% trifluoroacetic acid) and left for air-drying at room temperature for 5–10 min. At least eight technical replicates were included for each *Dickeya* spp. isolate.

The MALDI-TOF MS spectra were acquired in linear positive ion mode at a laser frequency of 60 Hz across a mass/charge ratio (m/z) of 2000 to 20,000 Da using the Microflex LT/SH system with flexControl 3.4 software (Bruker Daltonics GmbH, Bremen, Germany). One μl of Bruker Bacterial Test Standard, which enables calibration over a mass range of 4 to 17 kDa, was applied on the MSP 96 target plate during each session of the recordings of the MALDI-TOF MS spectra. Each spot, corresponding to a single replicate per bacterial strain, was analysed three times, resulting in acquisition of 24–27 single spectra for each *D. solani* strain. MALDI-TOF MS spectra for *Dickeya lacustris* LMG 30899^TS^ and *Dickeya aquatica* LMG 27354^TS^ were acquired and processed in an identical manner.

#### Local database construction

The raw MALDI-TOF MS data were analysed with flexAnalysis 3.4 software. Automatic pre-processing using default programme options included such steps like baseline correction to remove the effect of the noise introduced by the matrix, filtering to smoothen the signal, with alignment of all spectra using common m/z and automatic peak detection on average spectra (area and/or intensity). All spectra were visually checked and manually edited in terms of flat-line, outliers, and the presence of single spectra with peaks differing from the other spectra, leading to removal of the questionable spectra. At least a total of 20 pre-processed spectra per each *D. solani* strain were used to calculate the strain Main Spectrum Profile (MSP) using the automated function of MALDI Biotyper Compass Explorer Module 4.1 (Bruker Daltonics GmbH, Bremen, Germany). Additionally, a total of 449 pre-processed *D. solani* spectra were used to create a reference MSP for the *D. solani* species by extracting the information on the peak mass, peak frequency and peak intensity distribution. The classification of all strains was verified by creating the MSP dendrogram using the MALDI Biotyper Compass Explorer software v. 4.1.80 (Bruker Daltonics GmbH, Bremen, Germany). The principal component analysis (PCA) was performed with Bruker Daltonics proprietary software (MBT Compass Explorer module) using the default options.

### Virulence-associated features of *D. solani* strains

To attribute the virulence potential to the included *D. solani* strains (Table 1), *D. solani* IFB0099 and IFB0223, revealed in our former studies as either high or low virulent, respectively, were utilized as controls^20,30^.

All strains, which were stored at − 80 °C in the collection of IFB UG & MUG, have been plated on TSA and then cultured for 24 h at 28 °C. Five ml of Trypticase Soy Broth (TSB) were inoculated with a single bacterial colony collected from a TSA plate and incubated for 24 h at 28 °C with 120 rpm shaking. The overnight culture was centrifuged at 4779 g for 10 min. After discarding the supernatant, the bacterial pellet was washed twice and suspended in sterile 0.85% NaCl. Next, the density of bacterial suspension was adjusted to 0.5 in the McFarland (McF; approx. 10^8^ CFU ml^−1^) scale with a DEN-1B densitometer (Biosan, Riga, Latvia). The so-prepared 0.5 McF bacterial suspension was utilized in the below-listed evaluations.

#### Growth dynamics

The growth rate of *D. solani* strains was assayed in 96-well plates with the EnVision™ Multimode Plate Reader (PerkinElmer, USA). Ninety µl of TSB were inoculated with 10 µl of the 0.5 McF bacterial suspension. The absorbance at 600 nm was measured every 20 min during 24 h stationary incubation at 28 °C.

#### Bacterial ability to macerate plant tissues

The potato tissue maceration assay was conducted accordingly to Zoledowska et al.^32^ with slight modifications. Potato tubers cv. Lilly were rinsed in sterile distilled water, surface-sterilized for 10 min in 10% bleach (ACE, Poland), washed again in water and dried under the laminar flow cabinet for 30 min. The tubers were subsequently cut into approx. 1 cm slices, in which two–three holes of 0.5 cm in diameter were drilled per slice. The slices were placed in plastic boxes on the surface of a moistened Whatman filter paper and 50 μl of 0.5 McF bacterial suspension was used for the inoculation of each hole. Control slices were inoculated with 0.85% NaCl. The lid-covered boxes were incubated for 48 h at 28 °C. Afterwards, the diameters of the lesions were measured.

The chicory tissue maceration assay followed the protocol of Potrykus et al.^20^ with slight modifications. A hole was drilled with a pipette tip in a chicory leaf approx. 2 cm from its bottom. Five chicory leaves were placed on the surface of a moistened Whatman filter paper introduced into a sealed plastic bag. On each tip-drilled wound, 10 μl of the previously prepared 0.5 McF bacterial suspension was poured. After incubation for 48 h at 28 °C, the lengths of the lesions were measured. 0.85% NaCl was used as a negative control.

All the experiments were repeated three times, and each involved five technical replicates.

#### Plant cell wall degrading enzymes activities

PCWDEs activities were assessed as described in Zoledowska et al.^32^ with slight modifications.

For pectinase activity, 2 µl of the 0.5 McF bacterial suspensions were spotted on a M63 Y PGA medium. After 48 h incubation at 28 °C, the medium was flooded with 10% copper acetate. The halo zones around bacterial colonies corresponding to PGA degradation capacity were measured.

For cellulase activity, 2 µl of the 0.5 McF bacterial suspensions were spotted on M63 Y CMC plates. After 48 h incubation at 28 °C, the medium was covered with 1% Congo red solution and subsequently destained for 5 min with 1 M NaCl. The diameters of the halo zones around bacterial colonies interpreted as CMC degradation ability were measured.

For protease activity, 2 µl of the 0.5 McF bacterial suspensions were introduced into Skim milk agar plates. After 24 h incubation at 28 °C, the diameters of the halo zones surrounding bacterial colonies, which referred to protease activity, were measured.

All the experiments were repeated in triplicate, and each one involved four technical repeats.

#### Plant tissue colonising potency

Abilities necessary for the studied pathogen to efficiently colonize plant host tissue were examined as described previously^33^ with slight modifications.

For swimming motility evaluation, 2 µl of the 0.5 McF bacterial suspensions were spotted on a semi-solid Nutrient Broth (NB) medium with 0.3% agar. After 24 h of incubation at 28 °C, the diameters of the bacterial colonies were measured.

In terms of swarming motility, 2 µl of the 0.5 McF bacterial suspensions were spotted on an NB medium solidified with 0.6% agar. After 24 h incubation at 28 °C, the diameters of the bacterial colonies were measured.

To investigate the ability of the pathogen to form biofilm, 10 µl of an overnight bacterial culture was added to a 1.5 mL reaction tube with 400 µl of M9 medium. After 16 h incubation at 18 °C without shaking, 70 µl of 1% crystal violet solution was poured into the tube and incubated for 20 min. The excess of the dye was removed by triple washing with distilled water. After drying the tubes in the open air, 600 µl of 96% ethanol was poured into the tube and mixed by pipetting. The absorbance of the resultant solution was measured at 585 nm with the EnVision™ Multimode Plate Reader (PerkinElmer, USA). The absorbance of the blind samples was subtracted from the bacteria-containing samples. *P. aeruginosa* IFB9036 (PAO1) and *P. fluorescens* IFB9035 (CCM2115) strains were included as controls of the potent biofilm producers.

To establish bacterial ability to secrete siderophores, 2 µl of the 0.5 McF bacterial suspensions were spotted on CAS agar plates. After 24 h incubation at 28 °C, the diameters of the halo zones around bacterial colonies reflecting siderophore activity were measured.

All experiments were repeated three times, and each involved four technical replicates.

#### Biochemical profiles

The metabolic properties of *D. solani* strains (Table 1) in addition to *D. dadantii* 3937 were investigated with API 20 E (Biomérieux, France) according to the manufacturer’s guidelines. The following features have been examined: the production of β-galactosidase, arginine dihydrolase, lysine decarboxylase, ornithine decarboxylase, urease, tryptophan deaminase, gelatinase, H_2_S, acetoin and indole, in addition to the utilisation of citrate, glucose, mannitol, inositol, sorbitol, rhamnose, saccharose, melibiose, amygdalin and arabinose.

Nitrate Broth (Biomaxima, Poland) and Lactose-gelatine (Biomaxima, Poland) media were inoculated with the investigated bacterial strains to reveal their abilities to reduce nitrates in addition to state whether they ferment lactose and degrade gelatine, respectively. These media were examined after 24 h incubation at 28 °C, further monitored for over 72 h and interpreted according to the manufacturer’s guidelines.

The oxidase activity of *D. solani* strains (Table 1) in addition to *D. dadantii* 3937 was tested with OXItest (Erba Mannheim, Germany) according to the manufacturer’s instructions.

#### Statistical analysis and data visualisation

Statistical analysis and phenotypic data visualisation have been conducted with R 3.1.3^34^ in Rstudio v. 1.0.136. Preferably, tools from the *agricolae* package were applied. Levene’s test was implemented for evaluation of the equality of variances. Normality of the data was assayed with Shapiro–Wilk’s test. If the requirements for parametric analysis were fulfilled, the statistical significance between the means was stated by the analysis of variance (ANOVA) followed by Tukey’s honest significance test. Otherwise, the non-parametric Kruskal–Wallis test and the subsequent *post-hoc* test applying Fisher’s least significant difference (LSD) criterion were utilized. *p*-value < 0.05 was used for all the calculations. The final figures have been assembled in 0.92.3 Inkscape (GNU General Public License, USA).

## Results and discussion

### Phyloproteomic analysis on *D. solani* strains

Our former research on comparative genomics in *D. solani*^29,30^ added to knowledge on the high genomic uniformity in this taxon^11^ although it did not yield conclusive answers explaining phenotypic variability in this species. In this view, we herein implemented MALDI-TOF MS for analysing biodiversity among *D. solani* strains at the proteome level.

**Figure 1:**
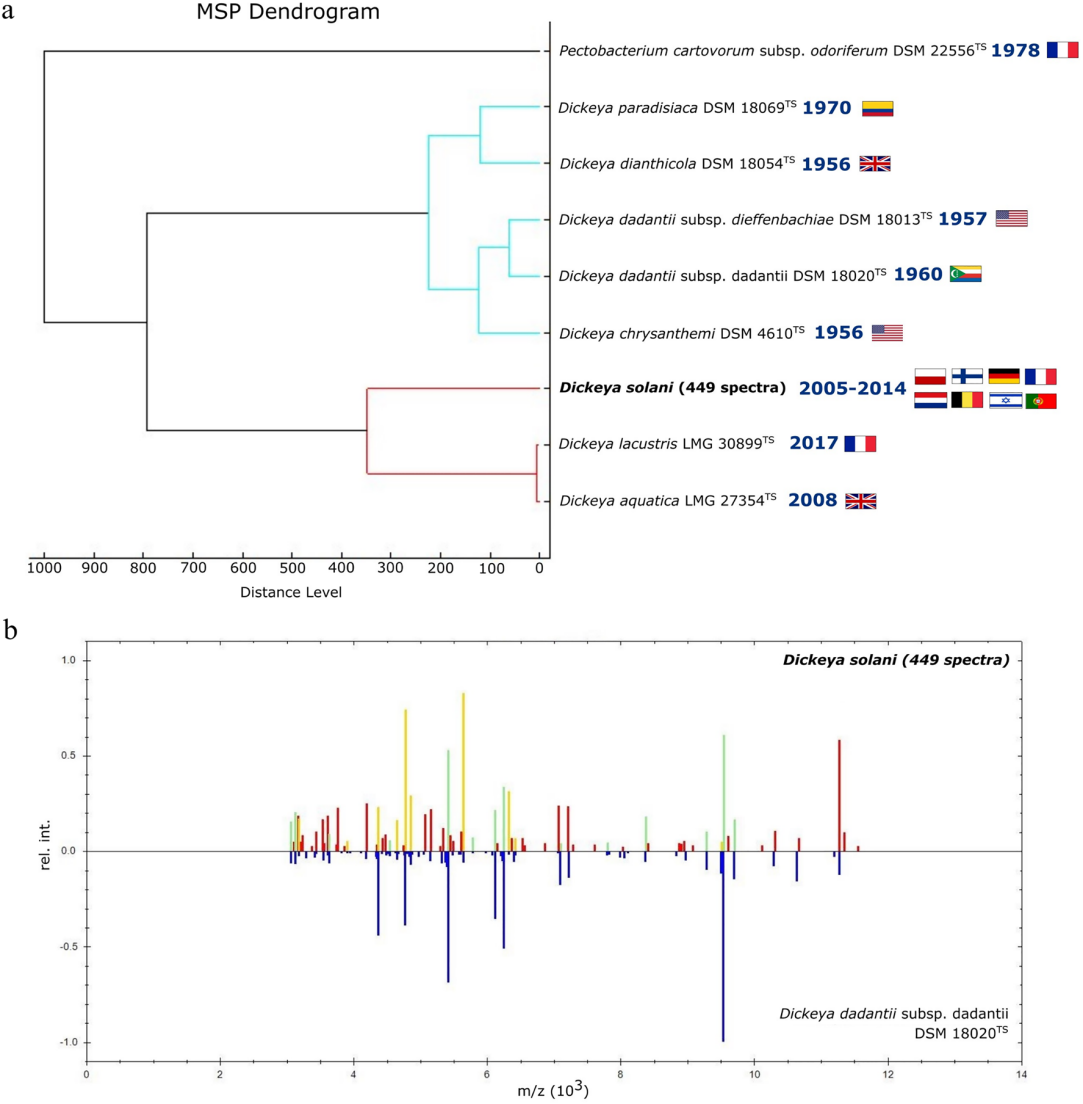
MALDI Biotyper-cluster analysis showing a dendrogram of the MSP generated for 20 *D. solani* strains in contrast to other *Dickeya* spp. The closeness of the relationships between the studied microorganisms is reflected by arbitrary distance levels normalized to a maximum value of 1000. 449 MALDI-TOF MS spectra of *D. solani* strains were registered for this analysis (**a**). Integration of the main protein spectra generated MSP for 20 *D. solani* strains and the *D. dadantii* DSM 18020^TS^ MSP, which is the closest match in the Bruker database (log score 1.720), revealed a set of common and unique peaks with the corresponding mass-to-charge (m/z) and relative intensities. Blue peaks derived from the reference *D. dadantii* MSP are displayed using an inverted intensity scale. The colour of the peaks reflects the degree of matching with the reference MSP; green bars representing a full match, yellow—a partial match, and red—no match (**b**).

The performed cluster analysis using the MALDI Biotyper primarily revealed that the main spectra of the 20 studied *D. solani* strains formed a distinct branch separate from the Main Spectra Profiles (MSPs) of other *Dickeya* species (see Fig. 1a). This finding is of high importance for to-species identification of *D. solani* and confirms the data of Šalplachta et al.^35^. Here, in the MSP dendrogram generated via hierarchical clustering, two main separate clades involving *Dickeya* spp. could be noted. The first clade included all the analysed *D. solani* strains (basing on 449 registered spectra), grouping separately from a sister clade containing *D. lacustris* LMG 30899^TS^ and *D. aquatica* LMG 27354^TS^. The other main clade of the dendrogram comprised an ingroup with two representatives of the subspecies of *D. dadantii* (*D. dadantii* subsp. *dadantii* DSM 18020^TS^, *D. dadantii* subsp. *dieffenbachiae* DSM 18013^TS^), a sister group with *D. chrysanthemi* DSM 4610^TS^, which all shared a node with a sister clade comprising *D. paradisiaca* DSM 18069^TS^ and *D. dianthicola* DSM 18054^TS^ (Fig. 1a). The reference MSP spectra for the listed Type Strains are included in the proprietary Bruker’s MBT Compass Library (Revision H, 2021. Ref. 1,829,023). The calculated normalized distance levels suggested that the obtained whole-cell intact proteins profiles of *D. solani* strains differ substantially from the MALDI-TOF spectra acquired for the other representatives of *Dickeya* species, which supports the applicability of this method for to-species classification of the environmental isolates. The comparison of the MSP generated for the 20 analysed *D. solani* strains (449 in total) and the *D. dadantii* DSM 18020^TS^ MSP, which is the closest match in the Bruker database (log score 1.720), indicated a set of common and unique peaks with the corresponding mass-to-charge (m/z) and relative intensities (Fig. 1b). The latter data can be useful for differentiation between these two SRP species and agree with Šalplachta et al.^35^.

**Figure 2:**
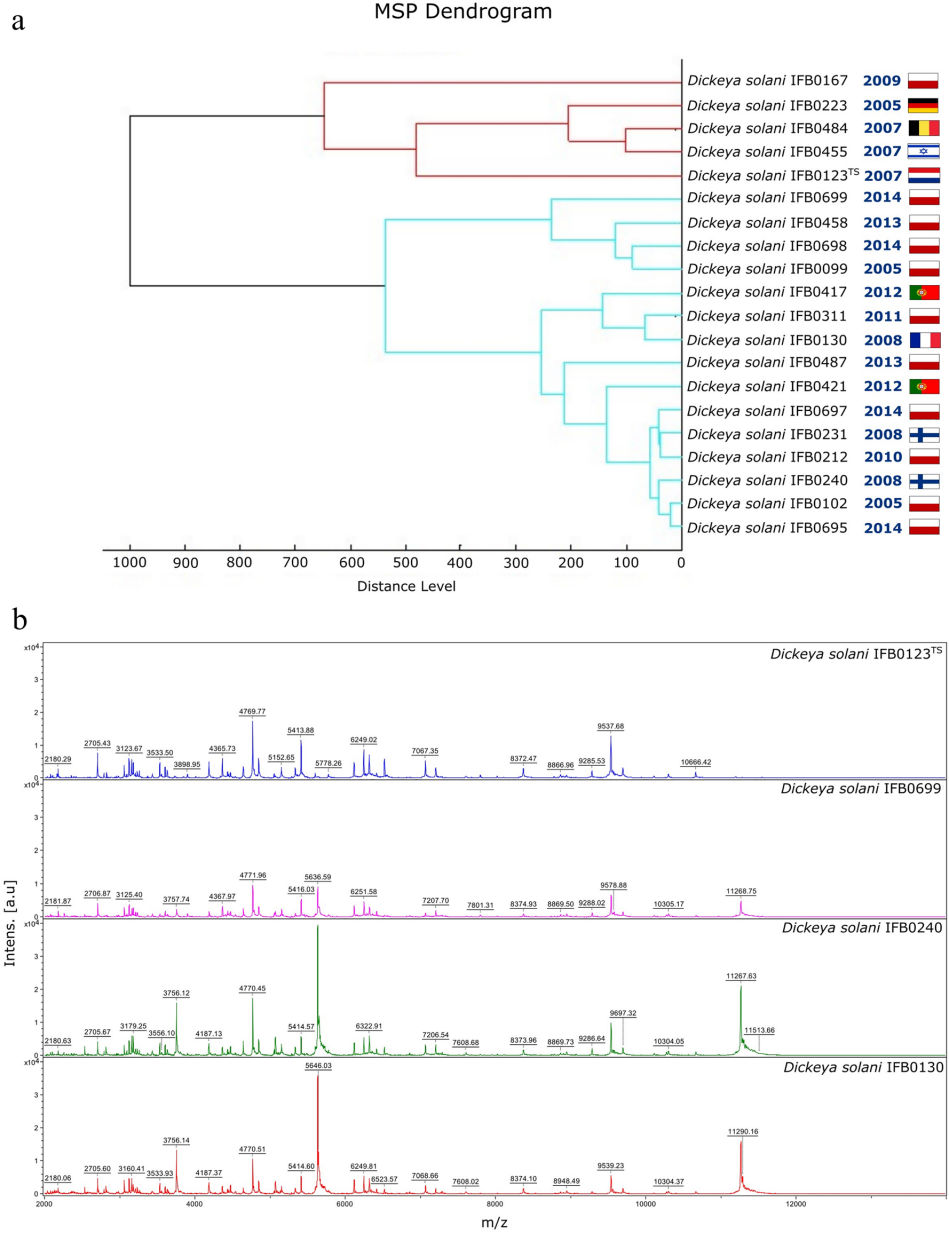
MALDI Biotyper-cluster analysis of 20 *D. solani* strains visualized as a dendrogram of the MSP that revealed intraspecies variation in the MALDI-TOF MS intact protein profiles among the studied isolates. The closeness of the relationships between the investigated microorganisms is reflected by arbitrary distance levels normalized to a maximum value of 1000 (**a**). Comparison of the MALDI-TOF MS representative spectra, as selected based on a PCA analysis, of four *D. solani* strains described in this study. The top panel shows the MALDI-TOF MS spectrum registered for *D. solani* type strain IPO2222^TS^ (IFB0123); the following representative spectra were acquired for *D. solani* IFB0699, IFB0240 and IFB0130. The assigned masses are depicted in Da (**b**).

Interestingly, in this research for the first time the MALDI Biotyper-cluster analysis was applied to reveal intraspecies differences in the whole-cell protein MALDI-TOF MS profiles among 20 *D. solani* strains (Fig. 2a). In the computed cladogram three main clusters have been observed. In the first clade from the top, an ingroup with *D. solani* IFB0484 (isolated in Belgium) and IFB0455 (from Israel) shares a node with IFB0223 (isolated in Germany). These three strains cluster within the same group with the *D. solani* type strain IPO2222^TS^ (IFB0123) (from the Netherlands) that has a sister group with a single leaf IFB0167 (isolated in Poland). The second clade solely contains strains isolated in Poland. IFB0698 and IFB0099 cluster most tightly and share the same node with IFB0458. The so-organized group has a sister taxon with a single tip formed by IFB0699. The third main cluster comprises the remaining 11 *D. solani* strains. Here, the lowest distance based on MALDI-TOF MS profiles was noted between IFB0102 and IFB0695. Interestingly, these isolates both originate from Poland, though they were isolated either in 2005 or 2014, respectively. The latter two strains form a single group with IFB0240 (from Finland) and this whole clade has a sister group comprising most closely related IFB0212 (isolated in Poland) and IFB231 (from Finland) in addition to IFB0697 (isolated in Poland). The clade with the above-listed six *D. solani* strains shares a common node at first with IFB0421 (from Portugal) and subsequently with IFB0487 (from Poland). The organized in such a way single cluster possesses a sister group including most closely related *D. solani* IFB0311 (isolated in Poland) and IFB0130 (from France) grouped together with IFB0417 (isolated in Portugal) as depicted in Fig. 2a. Importantly, four MALDI-TOF MS fingerprints, showing visible differences in the peak masses, peak frequencies and distribution of peak intensities, were pointed out as representative by PCA for the herein studied *D. solani* that grouped into diverse clusters of the MALDI Biotyper dendrogram (Fig. 2b).

To the best of our knowledge, solely van der Wolf et al.^11^ and Šalplachta et al.^35^ subjected *D. solani* strains to MALDI-TOF MS analyses before. Šalplachta et al.^35^ observed characteristic fingerprints allowing for the reliable differentiation and classification of *D. solani* strains with the MALDI-TOF MS, though they did not report any intraspecies variability in this taxon. Van der Wolf et al.^11^ described a set of 24 masses as discriminatory for *D. solani* species and underlined tight grouping within this taxon, although in the there-presented dendrogram of the whole-cell MALDI-TOF MS fingerprints, two clades enclosing 5 and 3 *D. solani* strains, respectively, could have been observed.

Here, we confirmed that by recording MALDI-TOF MS spectra, *D. solani* strains can be easily differentiated from the other *Dickeya* spp., which may contribute to the broader usage of MALDI-TOF MS as a fast diagnostic tool for routine to-species identification of SRP. The current report on four representative MALDI-TOF MS fingerprints for *D. solani* strains adds to knowledge on the characteristics useful for the identification of this species. Previously, solely the MALDI-TOF MS protein mass fingerprint for the Type Strain *D. solani* IPO 2222^11^ and species-specific MALDI-TOF mass signals^35^ in addition to a single representative MALDI-TOF^35^ mass spectrum for an unspecified *D. solani* strain were described.

Furthermore, MALDI-TOF MS technology has been herein applied for a phyloproteomic study on the largest pool of 20 *D. solani* strains. By these means, intraspecies variation in the whole-cell mass fingerprints within this highly homogenous taxon has been revealed for the first time. In this view, it is worthwhile to consider subtle deviations among *D. solani* strains deciphered by alternative approaches. Khayi et al.^36^ noted variabilities among other 20 *D. solani* strains in MLSA computed on 11 concatenated housekeeping genes, *i.e. gapA*, *fusA*, *rpoD*, *gyrB*, *rplB*, *purA*, *gyrA*, *recA*, *rpoS*, *dnaX* and *dnaA*. Also, whole genome-based comparisons^29^ pointed to minor differences between *D. solani* strains. Even though the calculated ANIb, ANIm and Tetra values are enclosed in 98.55–100, 98.71–99.98 and 0.99976–1.0 ranges^29^, respectively, the reports on high uniformity within this taxon dominate^1^.

### Virulence-associated features of *D. solani* strains

Observation of intraspecies variation at the level of whole-cell proteomes tempted us to look for a putative correlation with the virulence-associated features exhibited by 20 *D. solani* strains. In this study, we included *D. solani* IFB0099 and IFB0223 as the representatives of high and low virulent strains, respectively^20,30^.

#### Bacterial growth dynamics

Concerning that effective production of virulence determinants by SRP is dependent on the growth phase or the final population density^14^, we firstly investigated the bacterial growth dynamics. Not only *D. solani* IFB0223, reported before by Potrykus et al.^20^ to show a slower cell division rate than *D. solani* IFB0099, but also *D. solani* IFB0455, *D. solani* IFB0123 and *D. dadantii* 3937 reached significantly lower optical densities in vitro after 24 h incubation in a liquid TSB medium at 28 °C than the rest of the analysed isolates (Supplementary Fig. 1).

#### Plant tissue macerating potency

Subsequently, we investigated whether the observed divisions in the cell multiplication rates coincide with the virulence potential of *D. solani* strains.

**Figure 3:**
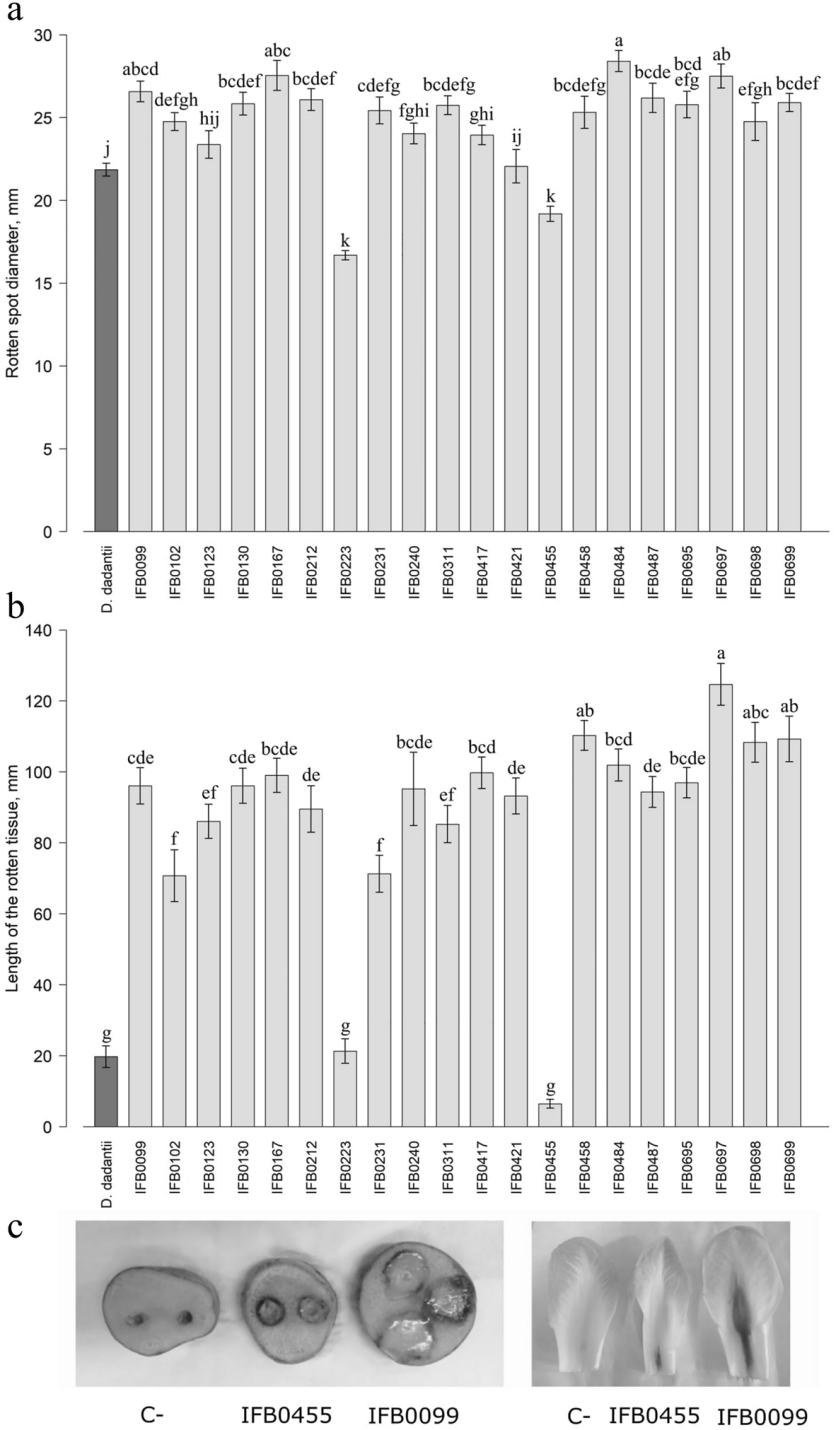
Plant maceration capacity of *D. solani* strains on potato tubers cv. Lilly (**a**) and chicory leaves (**b**). Means ± SE are shown. Means marked with the same letter are not significantly different at *p* < 0.05 according to the Kruskal–Wallis test followed by a *post-hoc* LSD. *D. dadantii* 3937 was included as an interspecies reference. (**c**): Exemplary photographs of the macerated plant tissue by the high and low virulent *D. solani* strains. C-: negative control.

The vast majority of *D. solani* strains showed a higher ability to macerate potato and chicory tissues than the reference *D. dadantii* 3937 strain (Fig. 3), which agrees with the previously suggested great devastating potential of *D. solani* species^11^. Just *D. solani* IFB0223 isolated in Germany in 2005 from potato rhizosphere, which finds confirmation in Potrykus et al.^20^, and *D. solani* IFB0455 obtained from seed potatoes imported to Israel from the Netherlands in 2007 exhibited significantly lower plant tissue maceration potencies on potato and chicory than the other *D. solani* isolates. *D. solani* IFB0484 obtained in Belgium in 2007, was the most effective in macerating potato tissue, while *D. solani* IFB0697 isolated in Poland in 2014 performed most potently on chicory leaves. Regarding strains impaired in triggering disease symptoms, either IFB0223 or IFB0455 (that also exhibited impaired cell multiplication potency; Supplementary Fig. 1) stood out, depending on the studied plant host (Fig. 3). Interestingly, IFB0123 that similarly to IFB0223, IFB0455 and *D. dadantii* 3937 reached lower optical densities during the stationary growth phase (Supplementary Fig. 1) macerated plant tissue with higher potency than the above-listed strains (Fig. 3).

#### Activities of plant cell wall degrading enzymes

**Figure 4:**
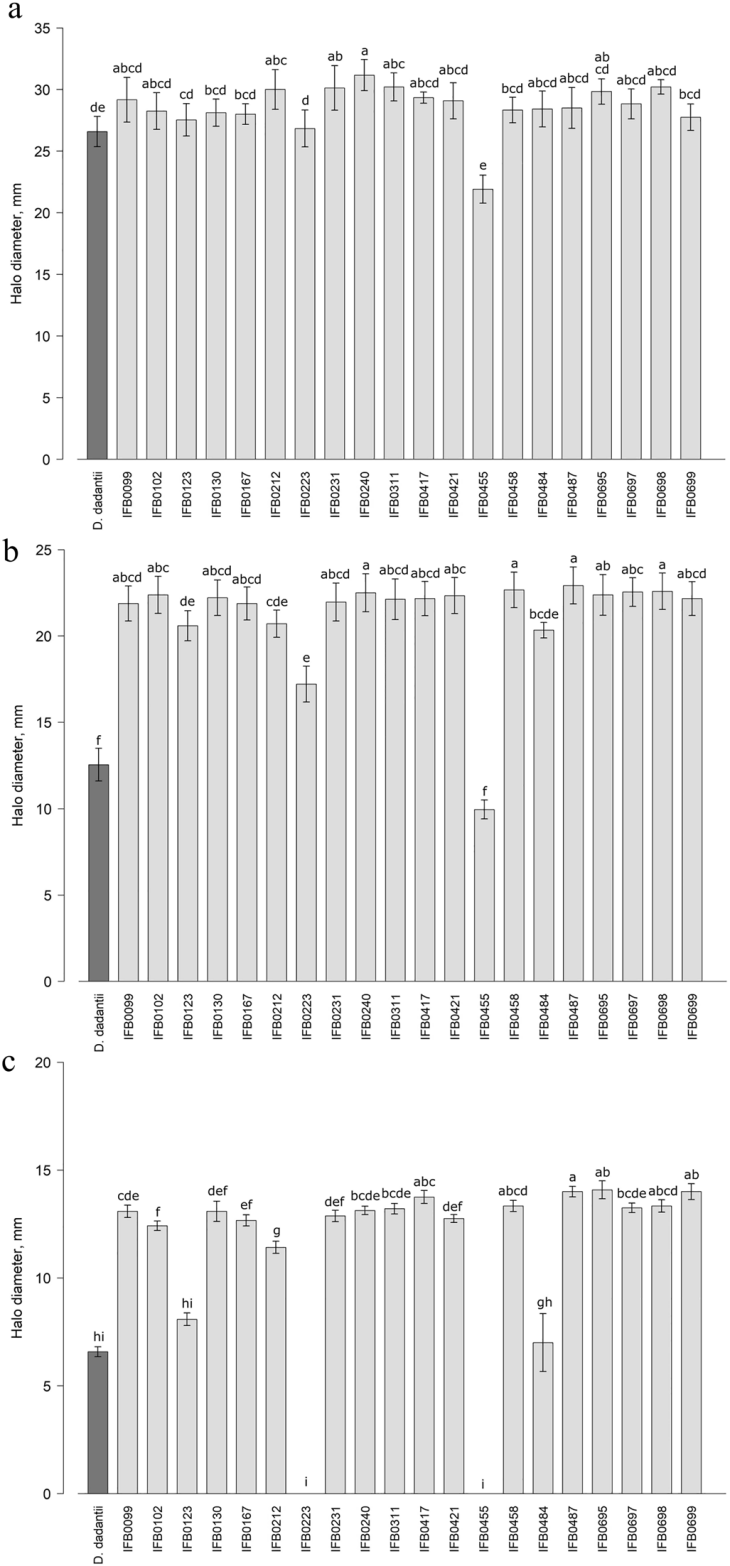
Plant cell wall degrading enzymes, *i.e.* pectinase (**a**), cellulase (**b**) and protease (**c**), activities of the studied *D. solani* strains. Means ± SE are shown. Means marked with the same letter are not significantly different at *p* < 0.05 according to the Kruskal–Wallis test followed by a *post-hoc* LSD. *D. dadantii* 3937 was included as an interspecies reference.

An ability to destroy plant tissue and successfully assimilate its intracellular components was associated in SRP with the effective production and secretion of PCWDEs^15^. Here, all the *D. solani* strains, except for IFB0455, presented a high, relatively uniform pectinase activity (Fig. 4a). A significantly lower effectiveness in PGA degradation (Fig. 4a) was shown by the *D. solani* IFB0455 strain, which was one of the least potent plant tissue macerators (Fig. 3) and exhibited an impaired cell multiplication rate (Supplementary Fig. 1). The interspecies reference *D. dadantii* 3937 showed the second lowest pectinase action among the tested strains (Fig. 4a). It is worth to acknowledge that IFB0223, of less efficient growth dynamics (Supplementary Fig. 1) and lower plant maceration potency (Fig. 3), did not stand out in pectinases production in a statistically significant manner from the other *D. solani* strains characterized (Fig. 4). As all the genomes of *Dickeya* spp. encode the majority of 6 protein secretion systems^1^, the intraspecies variation in the PCWDEs production might be rather linked with the presence/absence of the virulence-related genes or notable differences in their transcriptional networks. This hypothesis is supported by the fact that the expression of *pel* genes depends on multiple stimuli, *e.g.* the occurrence of pectin degradation products, cyclic adenosine monophosphate, bacterial growth phase, external temperature, pH, osmolarity, in addition to iron concentration^37^. In view of numerous factors affecting the production of pectinases, it does not surprise that Potrykus et al.^20^ observed a higher divergence in Pels activities, as determined by quantitative spectrophotometric measurements of the formation of unsaturated products from polygalacturonate, between the low virulent IFB0223 and the isolates of higher virulence (*e.g.* IFB0099), than it was noted in this work for the overall pectinase activity examined by the M63 Y PGA plate assay. As all the *pel* genes were present in the genomes of both high virulent (IFB0099) and low virulent (IFB0223) *D. solani* strains^30^, the search for explanations of this phenomenon continues.

In terms of cellulase activity, the vast majority of the investigated *D. solani* strains showed a notable activity of this group of enzymes (Fig. 4b) except for *D. solani* IFB0223, IFB0455 and the included interspecies reference *D. dadantii* 3937. This outcome agrees with Potrykus et al.^20^ and Golanowska et al.^28^ at the same time coinciding not only with the impaired growth multiplication rates (Supplementary Fig. 1), plant tissue maceration capacities (Fig. 3), but also other PCWDEs activities (Fig. 4).

The most outstanding variation among all the tested PCWDEs was shown in the ability of *D. solani* strains to produce proteases (Fig. 4c). No activity of this group of enzymes was observed for the low virulent *D. solani* IFB0223 (which corresponds with Potrykus et al.^20^) and IFB0455, while the *D. solani* IFB0123 (IPO2222^TS^), IFB0484 and *D. dadantii* 3937 reference strain revealed a significantly decreased protease activity in comparison to the other strains analysed (Fig. 4c). Even though an indispensable impact of proteases on the overall virulence of SRP has been questioned^19^, here the diminished plant tissue macerating potencies of *D. solani* IFB0223 and IFB0455 coincided with the lack of protease activity. These enzymes contribute to disruption of the plant cell wall and cellular membranes, which putatively not only allows for an access to the additional source of nitrogen, but also results in cleavage of the host resistance proteins^38^. As the genome of *D. solani* IFB0223 possesses all the proteases-encoding genes with 100% sequence homology to the genomes of 13 other *D. solani* strains^30^, either differences in the gene expression regulatory network or an insufficient basal level of active proteases to release inductive peptides necessary for triggering the process of proteolysis may be hypothesized, as suggested by Wandersman et al.^39^.

#### Other virulence determinants

**Figure 5:**
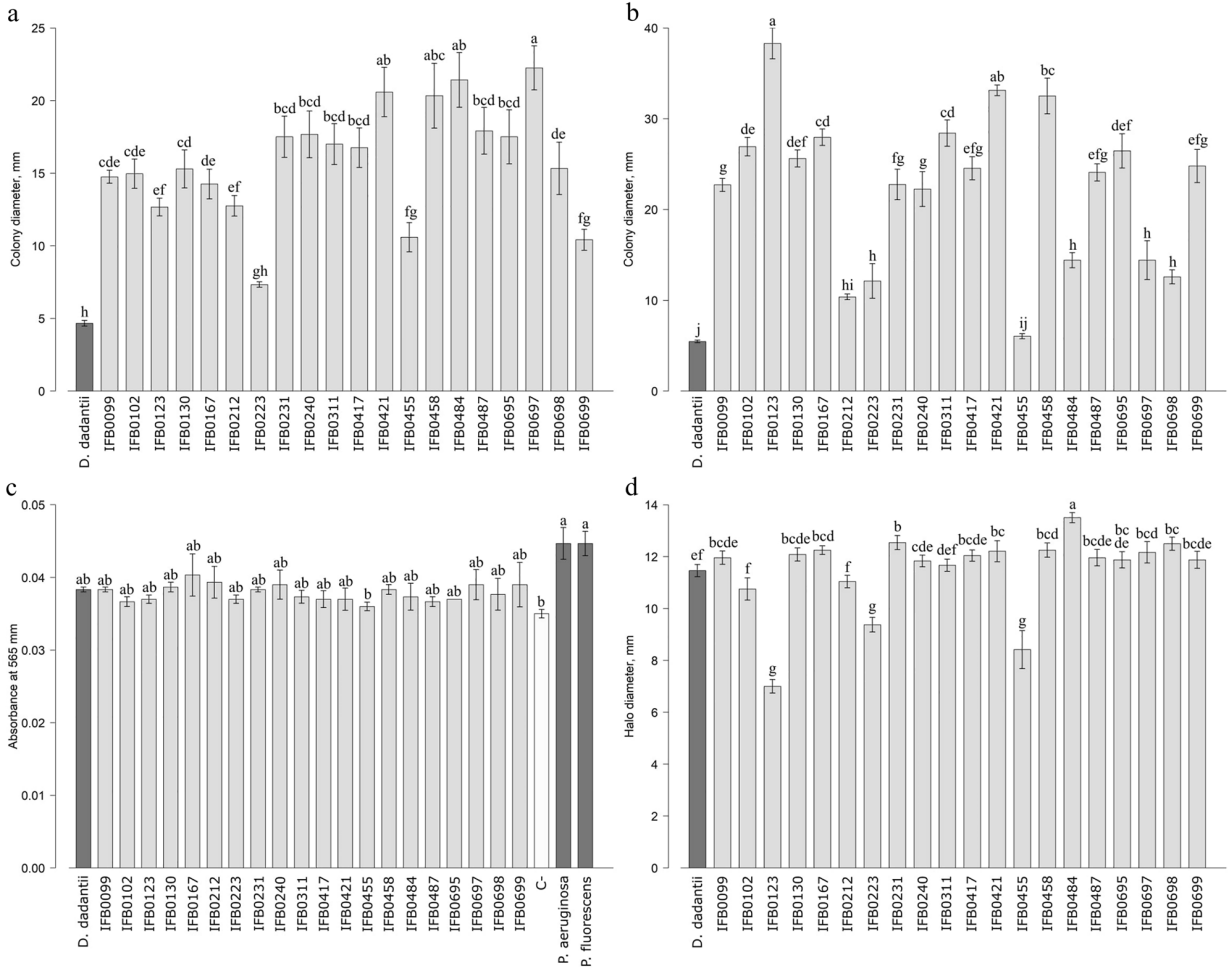
Other virulence determinants, *i.e.* swimming motility (**a**), swarming motility (**b**), biofilm formation capacity (**c**) and siderophores production (**d**), of the studied *D. solani* strains. Means ± SE are shown. Means marked with the same letter are not significantly different at* p* < 0.05 according to the Kruskal–Wallis test followed by a *post-hoc* LSD (**a**,**b**,**d**) or ANOVA followed by Tukey’s honest significance test (**c**). *D. dadantii* 3937 was included as an interspecies reference. *P. aeruginosa* PAO1 and *P. fluorescens* CCM2115 strains were included as controls representing potent biofilm producers. C-: negative control.

The tested *D. solani* strains differed considerably in their swimming capacities (Fig. 5a). Among the most efficient swimmers the IFB0421, IFB0458, IFB0484 and IFB0697 strains should be listed, while the least potent swimming was shown by *D. solani* IFB0223 (similarly to Potrykus et al.^20^), IFB0455 and *D. dadantii* 3937 (all three being impeded in their growth dynamics, plant tissue maceration capacities and PCWDEs production; Supplementary Fig. 1, Figs. 3, 4). Interestingly, the IFB0699 strain that should also be mentioned among inefficient swimmers (Fig. 5a) was at the same time able to degrade plant tissue in a quite effective manner (Fig. 3). Deviations in swimming of *D. solani* may be explained by the complex chemotactic abilities of this pathogen, dependent on 43 methyl-accepting chemotaxis proteins (MCPs)^17^. Here, even a higher variation between distinct *D. solani* isolates was noted in the case of swarming (Fig. 5b). Similarly to what was observed in the other conducted assays, the low virulent strains, namely *D. solani* IFB0223 and IFB0455 in addition to *D. dadantii* 3937, displayed a minor ability to swarm (Fig. 5b). However, regarding the rest of the strains analysed, the swarming potency (Fig. 5b) did not coincide with the plant maceration capacity (Fig. 3). As swarming strongly depends on the available carbon source and is induced *in planta*, the plate motility assays mimic only to a limited extent a real-life situation, even though they are sufficient *e.g.* to distinguish a wild-type *D. dadantii* 3937 from the motility-affected mutants in *cheB*, *cheW*, *cheY*, *cheZ*, or *motA*^40^. The HAI5 island, comprising genes involved in the synthesis of LPS O-antigen, was stated essential for the bacteria to swarm^25^. The so-far studied *D. solani* strains (IPO2222^TS^, IFB0099, IFB0158, IFB0223) and *D. dadantii* 3937 showed a homogeneous structure of O-antigen in LPS^41^, which for sure does not resolve the herein described intraspecies variation in the swarming of *D. solani*.

Subsequently, the biofilm formation capacity of *D. solani* strains was studied. Under the herein applied conditions based on culturing bacteria in an M9 minimal medium incubated at 18 °C, the included *D. solani* isolates generated biofilm with a rather uniformly minor effectiveness (Fig. 5c). No relations between the generation of biofilm and plant tissue degrading capacities (Fig. 3) were noted. It should be stressed that the included reference strains of potent biofilm producers, *i.e. P. aeruginosa* PAO1 and *P. fluorescens* CCM2115, showed a better capacity to form biofilm than all the analysed *D. solani* and *D. dadantii* 3937 strains (Fig. 5c). The efficient microbial adhesion was associated with physicochemical properties of the cells’ surface affected by bacterial growth rate, the utilized medium and the applied culture conditions^42^. In this view, it may be assumed that by performing a biofilm formation assay in a nutrient-rich TSB medium at a higher temperature, for instance 28 °C, greater differences among the investigated *D. solani* strains might have been revealed.

As a pivotal microelement Fe is the subject of an ongoing struggle between the invader and the host^14^, the ability of *D. solani* strains to secrete siderophores was studied. Most of the isolates did produce the iron-scavenging molecules in a quite consistent, efficient manner (Fig. 5d). Solely, the diminished potency to secrete siderophores was shown by two low virulent strains of impaired growth, namely *D. solani* IFB0223 and IFB0455, in addition to *D. solani* IFB0123 (Fig. 5d). Interestingly, the included interspecies reference *D. dadantii* 3937, in spite of the decreased plant maceration potency and cell multiplication rate, produced iron-scavenging molecules efficaciously (Fig. 5d). The herein described observation that *D. solani* IFB0223 and IFB0455 of the lowest pectinase activity were also impeded in siderophores production finds confirmation in the previous studies linking both iron acquisition and pectinases production with the control of a negative regulator Fur^43^.

#### Biochemical profiles

In view of former communications associating metabolic capacities of the pathogens with their virulence potential^14^, we investigated the abilities of 20 *D. solani* strains and *D. dadantii* 3937 to conduct 23 biochemical reactions (Supplementary Table 1). Twenty-two of the studied features turned out to be common among all the tested bacterial strains with the notable exception of gelatine hydrolysis (Supplementary Table 1). In more details, all the *D. solani* strains and *D. dadantii* 3937 were capable of producing β-galactosidase, nitrites from nitrates, in addition to indole from tryptophan. Furthermore, each of the investigated isolates was able to utilize glucose, citrate, mannitol, inositol, saccharose, rhamnose, melibiose, and amygdalin in addition to arabinose as a source of nutrients (Supplementary Table 1). On the contrary, the included *D. solani* strains and *D. dadantii* 3937 did not show any activities of arginine dihydrolase, lysine decarboxylase, tryptophan deaminase, ornithine decarboxylase, or urease in addition to cytochrome oxidase. Also, every investigated bacterial strain exhibited an incapability of producing H_2_S, acetoin, utilizing sorbitol and fermenting lactose (Supplementary Table 1). Solely the ability to hydrolyse gelatine discriminated the herein analysed *D. solani* strains. The majority (*i.e.* 15) of the isolates efficiently decomposed this mixture of proteins and peptides. Contrarily, the low virulent strains IFB0223 and IFB0455, were incapable of hydrolysing gelatine, which coincided with their lack of protease activity (Fig. 4c), their impaired growth in vitro (Supplementary Fig. 1), as well as the reduced capacity to macerate plant tissue (Fig. 3). *D. solani* IFB0123 and *D. dadantii* 3937 showed minor abilities to break down gelatine, while *D. solani* IFB0102 and IFB0212 liquefied the whole test medium over a longer period of time (> 72 h) than the other 15 potent gelatine degraders. The herein described nearly identical metabolic profiles of all the studied *D. solani* isolates and *D. dadantii* 3937 are in agreement with Slawiak et al.^44^, Czajkowski et al.^13^ and Palacio-Bielsa et al.^45^. Only one discrepancy with the literature data was noted, as in this study and Slawiak et al.^44^, each *D. solani* strain utilized arabinose in contrast to Czajkowski et al.^13^. Curiously, the herein reported intraspecies variability in gelatine degradation contested the observations of Palacio-Bielsa et al.^45^ on the sharing of this feature by each of the there-analysed *D. solani* strains.

In summary, two *D. solani* strains, *i.e.* IFB0223 and IFB0455, exhibited a lower plant tissue macerating capacity in addition to decreased activities of the majority of the studied SRP virulence factors. Interestingly, their MALDI-TOF MS intact protein profiles were similar, placing IFB0223 and IFB0455 strains in the same clade in the MALDI Biotyper analysis. However, it is important to note that a definitive link between pathogenicity and the recorded MALDI-TOF MS spectra could not be established in this study. The clade comprising low virulent *D. solani* IFB0223 and IFB0455 in the computed MALDI Biotyper-cluster analysis includes also *D. solani* strains of higher virulence potential, such as IFB0167, IFB0484 and the *D. solani* type strain IFB0123 In the realm of human pathogens, MALDI-TOF MS was already proven useful for differentiation between antibiotic-resistant vs. susceptible isolates^8,46^, as well as highly and low virulent strains^46,47^. Therefore, we anticipate that MALDI-TOF MS will find future applications in the field of plant protection, not only for reliable identification, but also for typing and detecting virulence markers among economically significant bacterial phytopathogens.

## Conclusions

In this study, for the first time, a MALDI-TOF MS-based phyloproteomic analysis has unveiled intraspecies variations among 20 *D. solani* strains differing in their virulence-associated features in addition to the country of origin and year of isolation. The mass spectra for the representatives of *D. solani* grouping into four delineated clades are presented. Importantly, *D. solani* strains can be easily differentiated from the other *Dickeya* spp. by using the in-house reference library of MALDI-TOF MS spectra, extended by the MSP of *D. solani* type strain IPO2222^TS^ (IFB0123), which offers potential for the wider adoption of this rapid diagnostic tool in routine *D. solani* identification. In addition, deviations in growth rate, plant tissue macerating potential and protease activities among the studied 20 *D. solani* strains were noted, in contrast to the high uniformity among the metabolic profiles, which diverged only in terms of gelatinases activity.

### Supplementary Information

Supplementary Information.

## Data Availability

Data that support the findings of this study are available from the corresponding author on reasonable request. The *D. solani* strains isolated in Poland and described in this study are at disposal in the Collection of Plasmids and Microorganisms (KPD), member of the European Culture Collections’ Organisation (ECCO) registered also at the World Date Centre of Microorganisms (WDCM) under no. 1084, which is located at University of Gdańsk (Poland). The full genomic sequences of a number of the herein characterized *D. solani* strains, *i.e.* IFB0099, IFB0167, IFB0212, IFB0223, IFB0231, IFB0311, IFB0417, IFB0421, IFB0487 and IFB0695, have been deposited by our research group in the GenBank database and are publicly available under the following accession nos. CP024711, CP051457, JABAON000000000, CP024710, CP051458, JABAOO000000000, CP051459, CP051460, JABAOP000000000, JABAOQ000000000, respectively.
