# Supplementary material for: Phyloproteomic study by MALDI-TOF MS in view of intraspecies variation in a significant homogenous phytopathogen Dickeya solani
Source: Sci Rep. 2023 Nov 1;13:18863. doi: 10.1038/s41598-023-46012-3 (PMC10620192; doi:10.1038/s41598-023-46012-3)
Supplement: Supplementary file 1 — Supplementary Information. [file 41598_2023_46012_MOESM1_ESM.docx]

Supplementary materials

**Phyloproteomic study by MALDI-TOF MS in view of intraspecies variation in a significant homogenous phytopathogen *Dickeya solani***

Agata Motyka-Pomagruk, Weronika Babinska-Wensierska, Wojciech Sledz, Anna-Karina Kaczorowska, and Ewa Lojkowska^*^

^*^Corresponding Author: Professor Ewa Łojkowska; Phone: +48 58 523 63 45; e-mail address: ewa.lojkowska@biotech.ug.edu.pl

**Supplementary Table 1.** Biochemical features of the studied *D. solani* strains and *D. dadantii* 3937.

| **Biochemical feature** | **IFB0099** | **IFB0102** | **IFB0123** | **IFB0130** | **IFB0167** | **IFB0212** | **IFB0223** | **IFB0231** | **IFB0240** | **IFB0311** | **IFB0417** | **IFB0421** | **IFB0455** | **IFB0458** | **IFB0484** | **IFB0487** | **IFB0695** | **IFB0697** | **IFB0698** | **IFB0699** | ***D. dadantii* 3937** |
| --- | --- | --- | --- | --- | --- | --- | --- | --- | --- | --- | --- | --- | --- | --- | --- | --- | --- | --- | --- | --- | --- |
| β-galactosidase | + | + | + | + | + | + | + | + | + | + | + | + | + | + | + | + | + | + | + | + | + |
| Arginine dihydrolase | - | - | - | - | - | - | - | - | - | - | - | - | - | - | - | - | - | - | - | - | - |
| Lysine decarboxylase | - | - | - | - | - | - | - | - | - | - | - | - | - | - | - | - | - | - | - | - | - |
| Ornithine decarboxylase | - | - | - | - | - | - | - | - | - | - | - | - | - | - | - | - | - | - | - | - | - |
| Citrate utilization | + | + | + | + | + | + | + | + | + | + | + | + | + | + | + | + | + | + | + | + | + |
| H_2_S production | - | - | - | - | - | - | - | - | - | - | - | - | - | - | - | - | - | - | - | - | - |
| Urease | - | - | - | - | - | - | - | - | - | - | - | - | - | - | - | - | - | - | - | - | - |
| Tryptophan deaminase | - | - | - | - | - | - | - | - | - | - | - | - | - | - | - | - | - | - | - | - | - |
| Indole production | + | + | + | + | + | + | + | + | + | + | + | + | + | + | + | + | + | + | + | + | + |
| Acetoin production | - | - | - | - | - | - | - | - | - | - | - | - | - | - | - | - | - | - | - | - | - |
| Gelatin hydrolysis^b^ | + | -/+ | --/+ | + | + | -/+ | - | + | + | + | + | + | - | + | + | + | + | + | + | + | --/+ |
| Glucose utilization | + | + | + | + | + | + | + | + | + | + | + | + | + | + | + | + | + | + | + | + | + |
| Mannitol utilization | + | + | + | + | + | + | + | + | + | + | + | + | + | + | + | + | + | + | + | + | + |
| Inositol utilization | + | + | + | + | + | + | + | + | + | + | + | + | + | + | + | + | + | + | + | + | + |
| Sorbitol utilization | - | - | - | - | - | - | - | - | - | - | - | - | - | - | - | - | - | - | - | - | - |
| Rhamnose utilization | + | + | + | + | + | + | + | + | + | + | + | + | + | + | + | + | + | + | + | + | + |
| Saccharose utilization | + | + | + | + | + | + | + | + | + | + | + | + | + | + | + | + | + | + | + | + | + |
| Melibiose utilization | + | + | + | + | + | + | + | + | + | + | + | + | + | + | + | + | + | + | + | + | + |
| Amygdalin utilization | + | + | + | + | + | + | + | + | + | + | + | + | + | + | + | + | + | + | + | + | + |
| Arabinose utilization | + | + | + | + | + | + | + | + | + | + | + | + | + | + | + | + | + | + | + | + | + |
| Lactose fermentation^b^ | - | - | - | - | - | - | - | - | - | - | - | - | - | - | - | - | - | - | - | - | - |
| Nitrate reduction^a^ | + | + | + | + | + | + | + | + | + | + | + | + | + | + | + | + | + | + | + | + | + |
| Cytochrome oxidase^c^ | - | - | - | - | - | - | - | - | - | - | - | - | - | - | - | - | - | - | - | - | - |

Metabolic profiling was achieved with Analytical Profile Index (API) 20E assay (Biomérieux, France) in addition to the supporting analyses in Nitrate Broth^a^, Lactose-gelatin medium^b^ and with the use of OXItest^c^. +: the strain exhibited the listed ability, -: the strain lacked the stated ability, -/+ and --/+: inconclusive outcomes discussed in the main text.

**
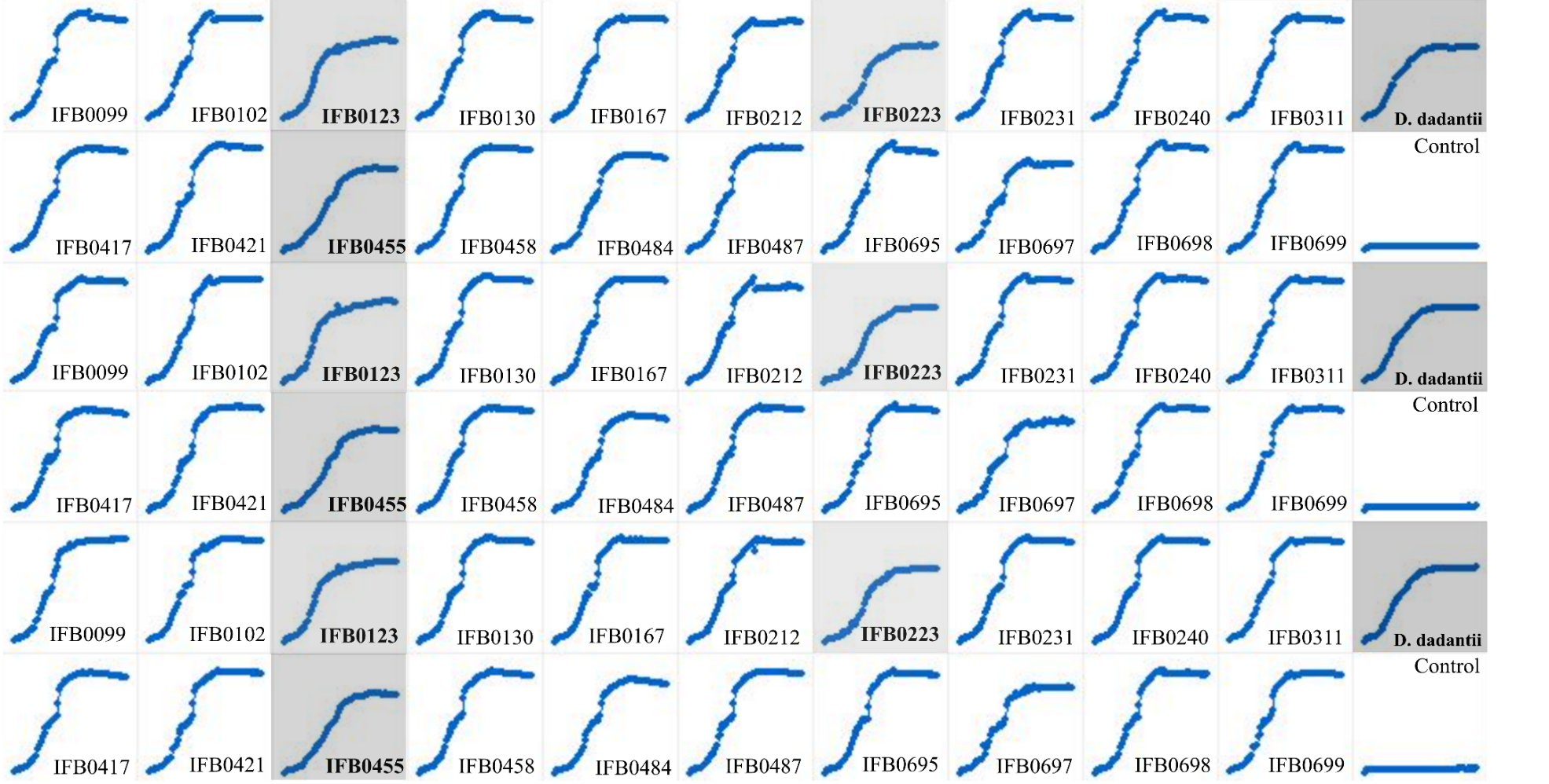
**

**Supplementary Figure 1.** Growth dynamics of *D. solani* strains in comparison to *D. dadantii* 3937.

*D. solani* IPO2222^TS^ (IFB0123), IFB0223, IFB0455 and *D. dadantii* 3937 that grew to significantly lower optical densities than the rest of the tested strains are colored in grey and their names are bolded. This experiment was done in triplicate. Control: uninoculated TSB medium. *D. dadantii* 3937 was included as an interspecies reference.
